# How Does the Starting Order in the First and Second Run Affect the Final Rank in the FIS World Cup Giant Slalom?

**DOI:** 10.3389/fspor.2022.858123

**Published:** 2022-07-06

**Authors:** Glenn Björklund, Mikael Swarén

**Affiliations:** ^1^Department of Health Sciences, Swedish Winter Sports Research Centre, Mid Sweden University, Östersund, Sweden; ^2^Swedish Unit for Metrology in Sports, Department of Sports, Fitness and Medicine, Dalarna University, Falun, Sweden

**Keywords:** alpine skiing, race tactics, performance analysis, winter sports, elite sports

## Abstract

The aim of this study was to determine the impact of runs 1 and 2 on overall rank in Giant Slalom. Data from 15 seasons (2005/2006–2019/2020) including and unique starts for women (*n* = 2,294) and men (*n* = 2,328) were analyzed. Skiers were grouped based on final ranks 1–3 (G3), 4–10 (G10), and 11–20 (G20) and separately analyzed for women and men. A Wilcoxon-signed rank test was used for comparisons between runs 1 and 2, while a multi-nominal logistic regression was used to identify odds ratios (OR) associated with group rank. Women had similar run times for runs 1 and 2 (*p* = 0.734), while men had faster times on run 2 (*p* < 0.001). The strongest association to G3 was during run 1 for run time (men: OR 1.06–1.12; women: OR 1.06–1.11, all *p* < 0.01) and gate-to-gate times (men: OR 33–475; women: OR 81–2,301, all *p* < 0.001). Overall, this study demonstrates the importance of a fast first run for improving the final ranking group and the need to increase the tempo going from the first to the second run for men.

## Introduction

The difference among Alpine World Cup (WC) skiers is often only a few hundredths of a second; however, there are shorter sections along the courses where the differences can vary by 10% (Supej and Cernigoj, 2006b; Supej et al., 2011). A detailed performance analysis can be obtained by comparing gate-to-gate times, which is the time taken to pass between each gate of the course (Supej and Cernigoj, 2006b; Swarén et al., 2021). This can be considered as the skier's tempo that changes depending on the steepness of the terrain, the skiing speed, the course setting, and the skier's skill level. There are two main strategies to achieve high-level race performance (i) continuously aim for short section times over several consecutive sections or (ii) target a high-velocity gain resulting in a shorter time in the subsequent section (Supej and Cernigoj, 2006b; Spörri et al., 2012, 2018).

Although Spörri et al. (2018) show it is of a higher priority to maintain skiing speed than shortening the center of mass path length, a bad snow surface is likely to affect both and ultimately have a large negative impact on performance overall. Previous studies have shown skiing performance can be affected by changing conditions of the ski courses, especially in steeper sections of the course, and therefore the start order can significantly influence the skier's final ranking (Supej et al., 2005; Lešnik et al., 2013). A Giant Slalom race (GS) in alpine skiing consists of two different runs whereby the top 30 skiers in the first run qualify for the second, with the final race result being the combined time of both. For the first run, the starting order is drawn based on skiers' ranking points set by the International Ski Federation (FIS) (Maisano et al., 2016). The top 30 skiers are then divided into three groups where the top seven GS skiers draw for the start numbers 1–7, the second group for the start numbers 8–15, and the third group 16–30 (FIS, 2019/2020). With a high starting number, previous skiers have already indented the snow surface thus it is often more difficult to choose and maintain an individual line through the course. Therefore, the starting order in the second run is reversed whereby the skier who finished 30th in the first run begins the second run.

As skiing performance is affected by starting order and changing ski course conditions, and the reversed start order in the second run should allow slower skiers in the first run to make up time. However, it is likely that the slower skiers in the first run are ranked lower and therefore are less skilled in comparison to those in the starting group 1–7. Nevertheless, the reversed starting order also provides a good opportunity to reduce the time differences among the fastest skiers, as well as those in the starting group who may have executed a poor first run.

Despite the obvious importance of final rank and overall performance in GS, the influence of the starting order in GS has not yet been investigated. Consequently, knowledge regarding the importance of the different starting groups, as well as of whether male and female skiers are similarly affected by the starting order is lacking. Hence, this study aims to analyze the associations between (i) the starting order and the race result and (ii) the likelihood to make up time in the second run after a poor first run among men and women competing in FIS World Cup in Giant Slalom over several seasons.

## Methods

All race data were obtained from the International Ski Federation's (FIS) datacenter, an open public domain (www.fis-ski.com). Specifically, data were collected from the top 20 skiers who completed the second run of a WC event in GS between seasons 2005/2006 and 2019/2020 (*n* = 15 seasons), with a total number of starts for women *n* = 3,129 and men *n* = 3,164. Based upon the final race results (run 1 + run 2), the number of unique starts for overall race position groups 1–3 (G3), 4–10 (G10), and 11–20 (G20) were in total *n* = 2,294 and *n* = 2,328 for women and men, respectively. Additionally, the number of starts in the analysis for women and men based on their FIS ranking start/bib groups, 1–7 (B7), 8–15 (B15), and 16–30 (B30) were *n* = 2,541 and *n* = 2,564, respectively.

### Statistical Analysis

Race data were pre-checked for normality using the Shapiro–Wilk test showing that none of the data regarding run time, total time, or gate-to-gate time conformed to normal distribution. Accordingly, a non-parametric Wilcoxon-signed rank test was used for comparisons of the run and average gate-to-gate times between runs 1 and 2 for women and men, separately. The average gate-to-gate time for each run was calculated as the total run time divided by the number of turning gates. A point biserial correlation coefficient (*r*_*pb*_) was used as effect size with small interpretive benchmarks *r*_*pb*_ < 0.10, medium *r*_*pb*_ > 0.11 to < 0.36, and large *r*_*pb*_ > 0.37 (McGrath and Meyer, 2006). Comparisons between the groups as final rank (G3, G10, and G20) and start/bib numbers (B7, B15, and B30) within separate runs using run time and gate-to-gate times were performed using a Kruskal–Wallis test with epsilon squared (ε^2^) for determination of the effect size. A Dwass–Steel–Critchlow–Flinger test was applied for pairwise comparisons if there was a global significance for the Kruskal–Wallis test. Furthermore, a multi-nominal logistic regression was used to identify the run times and gate-to-gate times associated between different rank groups (G3, G10, and G20), and an χ^2^ test of independence determined the differences between start groups according to BIB number (B7, B15, and B30) and the group rank (G3, G10, and G20) using Cramer's V (*V*) as the effect size. All statistical analysis was performed using *jamovi* (The jamovi project, 2020). Due to the skewness of the data, the results are presented as a median and interquartile range [IQR], odds ratios (OR) with confidence intervals (95% CI), or with mean values where appropriate. The α level was set to < 0.05.

## Results

### Run Time

#### Total

The women had similar run times for runs 1 and 2 (69.7 s [64.1–73.7] vs. 69.4 s [64.3–74.1], *z* = 2.33, *p* = 0.734, *r*_*pb*_ = 0.007), while the men decreased the run time in run 2 (75.0 s [70.8–78.7] vs. 73.9 s [70.5–78.0], *z* = 9.44, *p* < 0.001, *r*_*pb*_ = 0.249). For the final ranking, the likelihood of being placed in the top group G3 had the strongest association with a fast run 1 for both women and men (Table 1). In run 2, the relation was reversed with a faster run most likely in the G20 group (Table 1).

**Table 1:** Multi-nominal logistic regression for run times in Giant Slalom FIS World Cup 2005–2020.

**Group**	**OR**	**95% CI**	***p*-value**
**Women**
**G10–G3**
Run 1 (s)	1.066	1.022–1.110	=0.003
Run 2 (s)	0.952	0.915–0.991	=0.015
**G20–G3**
Run 1 (s)	1.115	1.071–1.160	<0.001
Run 2 (s)	0.923	0.889–0.959	<0.001
**Men**
**G10–G3**
Run 1 (s)	1.060	1.019–1.102	=0.004
Run 2 (s)	0.957	0.922–0.994	=0.023
**G20–G3**
Run 1 (s)	1.104	1.064–1.147	<0.001
Run 2 (s)	0.932	0.899–0.966	<0.001

*Run times (s) represent finishing time for each separate run; G3, place 1–3; G10 place 4–10; G20 place 11–20. Data are reported as odds ratio (OR) with a 95% confidence interval (95% Cl) an p-values. An OR > 1.0 along with the 95% CI above 1.0 indicate that the reference group (G3) is faster than G10 and G20. Conversely, if OR is <1.0 with a 95% CI below 1.0 suggest that G3 is slower compared to the other groups, i.e., G10 and G20*.

#### Run 1

There was an overall difference between ranked groups for the women's run times in run 1 [χ^2^_(2)_ = 17.50, *p* < 0.001, ε^2^ = 0.00763]. The G3 group was faster than G20 (68.6 s [63.0–72.2] vs. 69.9 s [64.4–74.1], *p* < 0.001), and G10 was faster than G20 (69.3 s [63.6–73.2] vs. 69.9 s [64.4–74.1], *p* = 0.032). In run 1, for the men, there was an overall difference between groups [χ^2^_(2)_ = 23.75, *p* < 0.001, ε^2^ = 0.01021] with G3 significantly faster than G20 (73.6 s [69.0–77.1] vs. 75.1 s [71.0–78.9], *p* < 0.001) and G10 faster than G20 (74.4 s [70.0–78.0] vs. 75.1 s [71.0–78.9], *p* = 0.009).

#### Run 2

No differences were found between the groups in run time for the second run for women [χ^2^_(2)_ = 3.07, *p* < 0.216, ε^2^ = 0.00134] or men [χ^2^_(2)_ = 3.06, *p* = 0.216, ε^2^ = 0.00132].

### Gate-to-Gate Time

The median number of turning gates was 50 [48–53] and 44 [42–48] for men and women, respectively. No significant difference regarding the number of turning gates between run 1 and run 2 was found for men or women.

#### Total

The women had similar median gate-to-gate times for runs 1 and 2 (1.54 s [1.49–1.60] vs. 1.55 s [1.50–1.59], *z* = −1.94, *p* = 0.560, *r*_*pb*_ = 0.012), while the men decreased their median gate-to-gate times in run 2 (1.49 s [1.44–1.56] vs. 1.48 s [1.44–1.53], *z* = 4.88, *p* < 0.001, *r*_*pb*_ = 0.157).

#### Run 1

For women, there was a difference in gate-to-gate times in run 1 for the ranked groups [χ^2^_(2)_ = 66, *p* < 0.001, ε^2^ = 0.029] with G3 having the shortest time compared to both G10 and G20 (1.50 s [1.46–1.57] vs. 1.53 s [1.48–1.59] and 1.54 s [1.50–1.60], both *p* < 0.001) and G10 having a shorter gate-to-gate time than G20 (*p* < 0.001). There was also an overall difference between groups in gate-to-gate time for men in run 1 [χ^2^_(2)_ = 52.7, *p* < 0.001, ε^2^ = 0.023], with G3 displaying a shorter time than both G10 and G20 (1.46 s [1.42–1.53] vs. 1.48 s [1.43–1.55] and 1.49 s [1.45–1.56], both *p* < 0.001). G10 also had a shorter gate-to-gate time than G20 (*p* < 0.001).

#### Run 2

In the women's second run there was an overall difference in gate-to-gate times for the ranked groups [χ^2^_(2)_ = 10.9, *p* = 0.004, ε^2^ = 0.00475]. The only pairwise difference was between G3 and G20 (1.53 s [1.49–1.58] vs 1.54 s [1.50–1.60], *p* = 0.006). For men, there was a difference in gate-to-gate times [χ^2^_(2)_ = 7.93, *p* = 0.019, ε^2^ = 0.00341], with G3 showing a shorter time compared to G10 (1.47 s [1.43–1.52] vs 1.48 s [1.44–1.53], *p* = 0.022).

Overall, a short gate-to-gate time in the first run was strongly associated with a final rank in G3 (Table 2). Although a shorter gate-to-gate time in run 2 showed a greater likelihood to be linked to G20 (Table 2), this association was not as strong compared to run 1 in relation to G3.

**Table 2:** Multi-nominal logistic regression for gate-to-gate times in Giant Slalom FIS World Cup 2005–2020.

**Group**	**OR**	**95% CI**	***p*- value**
**Women**
**G10–G3**
Gate-to-gate run 1 (s)	80.77	9.363–696.9	<0.001
Gate-to-gate run 2 (s)	0.272	0.033–2.189	=0.221
**G20–G3**
Gate-to-gate run 1 (s)	2,301	288–18,371	<0.001
Gate-to-gate run 2 (s)	0.115	0.015–0.861	=0.035
**Men**
**G10–G3**
Gate-to-gate run 1 (s)	33.08	4.229–258.7	<0.001
Gate-to-gate run 2 (s)	0.285	0.922–0.994	=0.281
**G20–G3**
Gate-to-gate run 1 (s)	474.6	65.36–3,446	<0.001
Gate-to-gate run 2 (s)	0.085	0.001–0.759	=0.027

*Gate to gate times (s) represent finishing time for each separate run; G3, place 1–3; G10 place 4–10; G20 place 11–20. Data are reported as odds ratio (OR) with a 95% confidence interval (95% Cl) an p-values. An OR > 1.0 along with the 95% CI above 1.0 indicate that the reference group (G3) is faster than G10 and G20. Conversely, if OR is <1.0 with a 95% CI below 1.0 suggest that G3 is slower compared to the other groups, i.e., G10 and G20*.

### BIB Group

For both women and men, the B7 group showed the greatest number of placing in G3, while the B30 showed the least numbers (Tables 3, 4, respectively).

**Table 3:** Number of placings for different starting groups in women's FIS World Cup Giant Slalom 2005–2020.

	**BIB Group**			
**Rank Group**	**B7**	**B15**	**B30**	
G3 (*n*)	261	65	22	348
G10 (*n*)	261	279	222	762
G20 (*n*)	133	273	500	906
Total (*n*)	655	617	744	2,016
[χ^2^_(4, N = 2, 016)_ = 491, *p* < 0.001, *V* = 0.349]				

*BIB group 7, BIB 1–7; BIB group 15, BIB 8–15; BIB group 30, BIB 16–30; G3, rank 1–3; G10 rank 4–10; G20 rank 11–20*.

**Table 4:** Number of placings for different starting groups in men's FIS World Cup Giant Slalom 2005–2020.

	**BIB Group**			
**Rank Group**	**B7**	**B15**	**B30**	
G3 (*n*)	257	61	26	344
G10 (*n*)	279	276	200	755
G20 (*n*)	134	290	525	949
Total (*n*)	670	627	751	2,048
[χ^2^_(4, N = 2, 048)_ = 508, *p* < 0.001, *V* = 0.352]				

*BIB group 7, BIB 1–7; BIB group 15, BIB 8–15; BIB group 30, BIB 16–30; G3, rank 1–3; G10 rank 4–10; G20 rank 11–20*.

There was an overall difference in run time between different BIB groups for the women [χ^2^_(2)_ = 12.0, *p* = 0.002, ε^2^ = 0.00472], with B7 faster than both B15 and B30 (*p* = 0.020 and *p* = 0.003) and with no differences between B15 and B30 (*p* = 0.878). In run 2, for the women, there was no difference between the BIB groups in the run time [χ^2^_(2)_ = 2.28, *p* = 0.320, ε^2^ < 0.0001). Within the women's BIB groups, there was a change in ranking between runs 1 and 2 among all groups [χ^2^_(2)_ = 272, *p* < 0.001, ε^2^ = 0.107]. Two groups lost in ranking with the BIB 7 losing most places −5 (IQR = −12 to 0) and BIB 15 dropping by −3 (IQR −10 to 3), respectively. The only women's group that gained in ranking from the 1 to 2 run was BIB 30 with 2 (IQR = −4 to 9).

In the men's race, an overall difference was shown between different BIB groups [χ^2^_(2)_ = 19.78, *p* < 0.001, ε^2^ = 0.00772], with B7 being faster than both B15 and B30 (*p* = 0.011 and *p* < 0.001). No differences between B15 and B30 were shown (*p* = 0.368). In run 2, there was no effect found in the BIB group on run time for the men [χ^2^_(2)_ = 1.52, *p* = 0.467, ε^2^ < 0.0001). The change in ranking between the runs 1 and 2 differed within the men's BIB groups [χ^2^_(2)_ = 344, *p* < 0.001, ε^2^ = 0.134]. Two groups lost in ranking were BIB 7 losing most in ranking −7 (IQR = −15 to −1) and BIB 15 with a drop by −4 (IQR −11 to 4), respectively. The only group that gained in ranking was BIB 30 with 2 (IQR = −4 to 10).

## Discussion

This is the first study to report the importance of a fast first run and its strong association with the final ranking in GS events. The first run was found to be equally as important for a final rank for both women and men. Even though a fast second run was most likely to be associated with a lower-ranked group, it did not have the same impact on the final ranking as the first run. The gate-to-gate time and run time remained unchanged between the first and second runs for the women but decreased for the men, potentially showing a need for faster tempo in the second run in general. In addition, a lower BIB number showed greater occurrence in a higher ranking, for both women and men.

### Run Time

Previous research on performance profiling in GS has mainly focused on biomechanical aspects using a limited number of skiers (*n* < 15) (Lešnik et al., 2013). As the first study to include data from both sexes spanning over several seasons, the current results demonstrate how critical a fast first run is for final ranking in both women and men. The association between a fast first run was evident to be placed in G3 compared with the other two other groups. Furthermore, these results suggest that it is difficult to change group rankings after run 1. In a previous study (Supej and Cernigoj, 2006a), looking at performance within one specific race during two seasons using a small sample size (*n* = 6), the main outcome was to focus first on technical rather than the tactical aspects. Interestingly, while mainly studying run 1, the authors pointed out that the four fastest skiing times in run 2 were from skiers placed outside the top 20 in the first run. The reversed starting order in run 2 provides the slower skiers the opportunity to ski on a freshly prepared slope. However, as identified by the current study, the reversed starting order in run 2 does not appear as favorable as the skiers do not make up for the time lost in run 1 to climb to a better-ranked group in the final race result. It can be argued that the slower skiers from run 1 are less skilled and lower-ranked, which might explain why the early starting skiers in run 2 cannot make up the time lost from run 1. However, even highly ranked skiers in B7 and B15 who skied poorly in run 1 and started early in run 2, cannot benefit enough from the fresh conditions to make up for the time lost in run 1. Still, there are normally several skiers who improve their ranking between run 1 and run 2, but for top-ranked skiers, the current results show the importance of performing a fast run 1 and that it is unlikely to make up for lost time from the first run by skiing fast in run 2. Hence, even top-ranked skiers are unlikely to move to a better-ranked group, e.g., from G20 in run 1 to G3 in final race results, by skiing fast in run 2.

### Gate-to-Gate Time

Both women and men showed faster gate-to-gate times for G3 compared to G10 and G20 in run 1 and run 2, even though there is no difference in the number of turning gates between run 1 and run 2. It can therefore be argued that the reversed staring order in run 2 evens out the advantage by starting early in run 1. Still, the B7 group for men and women has the highest occurrence of G3, which suggests that the skiers in B7 have better skills compared to B15 and B30. However, it is difficult to explain why skiers in, e.g., B7 who finish in G20 after run 1 cannot make up for enough lost time to finish in G10 or G3 overall by performing a fast run 2. In theory, this should be possible, but it would require that they ski poorly enough in run 1 to start among the first seven in run 2, which rarely happens. Also, even though run 2 is a newly prepared course, tracks from run 1 exist on the slope, which means that the fresh conditions in run 2 are not as beneficial as in run 1. It can also be hypothesized that skiers might perform slightly better in run 2 as they are familiar with the snow and conditions from run 1. This could increase the skiers' individual velocity barriers, which would allow them to ski faster without making mistakes (Supej et al., 2011; Gilgien et al., 2020; Cross et al., 2021). Speculatively, B7 skiers might be better at benefiting from this phenomenon compared to B15 and B30 skiers. However, this is yet to be investigated.

### BIB Group

The frequency of numbers of skiers in the G3 group was evident for the skiers in the first BIB group (B7). Moreover, it was as common to end up in G3 and G10 for both women and men starting in B7 for run 1. While the B15 group that started between 8 and 15th place finished mostly in G10 and G20, indicating the importance of the start order, which provides the skiers with better snow conditions for optimal performance. This result is in accordance with previous results by Lešnik et al. (2013), despite them only investigating the first 15 skiers in run 1 during one European Cup slalom race. Still, the skiers in B7 are the seven best-ranked and are hence most likely to ski the fastest in run 1. However, run 2 has a reversed starting order, meaning that the fastest skiers in run 1 are starting last in the run 2. This gives the slower skiers from run 1 the opportunity to ski on a freshly prepared slope in run 2 and hence the best conditions to make up the lost time from run 1. Nevertheless, the slowest skiers from run 1 who qualified for run 2 still most often ended up in the lowest-ranked group, G20, with a ranking between 11 and 20, and the starting order did therefore not directly impact their final standings; there are other contributing factors. This suggests that the group that starts late in run 1 is not at an equal performance level as the skiers starting before them as these skiers still cannot make up time lost in during run 2 even after being provided optimal snow conditions. However, skiers obviously gain positions in run 2 also, but the present results show that these gained positions most often are within the group in which they started the run 2. Hence, it is unlikely for both men and women to gain enough positions to change the final position group compared to the position group after run 1, especially for B7 performing a poor run 1 and starting early in run 2.

### Comparison Between Women and Men

For the men, run 2 compared to run 1 has shorter run times with shorter gate-to-gate times. This is not the case for the women who have similar run times and gate-to-gate times in the first and second run. The reason for the faster pace of the men could be due to straighter courses with less traversing. In GS, Alejo et al. (2021) showed that male skiers benefit from skiing on a longer trajectory which allows them to maintain higher skiing speeds. However, female skiers do not seem to be able to maintain high enough skiing speeds when skiing on longer trajectories, which results in a worse ranking (Alejo et al., 2021). To avoid the tracks from run 1, it is likely that skiers are forced to use a longer skiing trajectory at different places in run 2. Compared to women, male skiers seem to be better at maintaining higher skiing speeds with longer trajectories which, together with an increase's velocity barrier, might explain why male skiers have a faster run 2 compared to run 1.

## Conclusion

This is the first study to report that the final group rank in GS is strongly related to a fast run 1. Even though run 2 favors the slower skiers from run 1, the favorable conditions in run 2 are not enough to reverse the final group rank. Lower start numbers and hence an early start order in run 1 seem favorable for the final ranking group. In the men's race, the faster gate-to-gate times in run 2 stress the importance of the ability to ski with a faster tempo to be in contention for a better final rank.

## Data Availability Statement

Publicly available datasets were analyzed in this study. This data can be found here: https://www.fis-ski.com/en/alpine-skiing.

## Author Contributions

GB and MS designed the study, performed the data analysis, interpreted the results, wrote the manuscript, revised the manuscript, approved the final version, and agreed to be accountable for all aspects of the study. All authors contributed to the article and approved the submitted version.

## Conflict of Interest

The authors declare that the research was conducted in the absence of any commercial or financial relationships that could be construed as a potential conflict of interest. The handling Editor TS declared a past co-authorship with the authors.

## Publisher's Note

All claims expressed in this article are solely those of the authors and do not necessarily represent those of their affiliated organizations, or those of the publisher, the editors and the reviewers. Any product that may be evaluated in this article, or claim that may be made by its manufacturer, is not guaranteed or endorsed by the publisher.
